# Supplementary material for: Orbitally dominated Rashba-Edelstein effect in noncentrosymmetric antiferromagnets
Source: Nat Commun. 2019 Nov 26;10:5381. doi: 10.1038/s41467-019-13367-z (PMC6879646; doi:10.1038/s41467-019-13367-z)
Supplement: Supplementary file 1 — Supplementary Information [file 41467_2019_13367_MOESM1_ESM.pdf]

SUPPLEMENTARY INFORMATION

Orbitally dominated Rashba-Edelstein effect in noncentrosymmetric antiferromagnets

Leandro Salemi,[1,*] Marco Berritta,[1] Ashis K. Nandy,[1,2] and Peter M. Oppeneer[1,†]

[1] *Department of Physics and Astronomy, Uppsala University, P. O. Box 516, S-751 20 Uppsala, Sweden*
[2] *Current address: School of Physical Sciences, National Institute of Science Education and Research, HBNI, Jatni 752050, India*

[*] leandro.salemi@physics.uu.se; [†] peter.oppeneer@physics.uu.se

Supplementary Figures

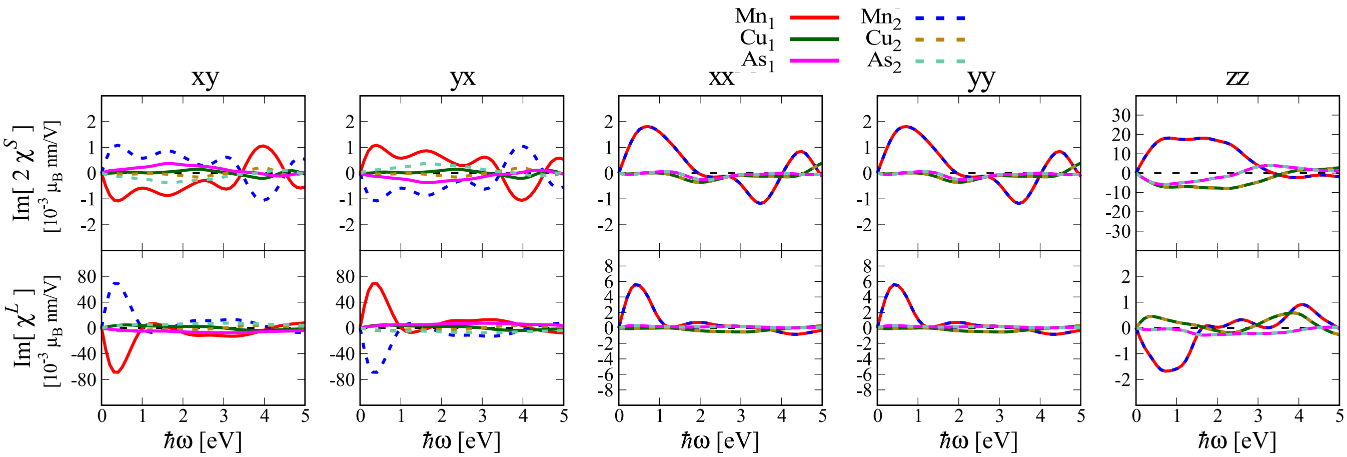

Supplementary Figure 1: Rashba-Edelstein tensors for antiferromagnetic CuMnAs with moments along the c-axis. Shown are the calculated imaginary parts of the nonzero elements of the spin and orbital Rashba-Edelstein tensors, $2\chi^S_{ij}(\omega)$ and $\chi^L_{ij}(\omega)$, i, j = x, y or z. Note that the imaginary parts of the spin and orbital responses are exactly zero for electric field frequency $\omega = 0$.

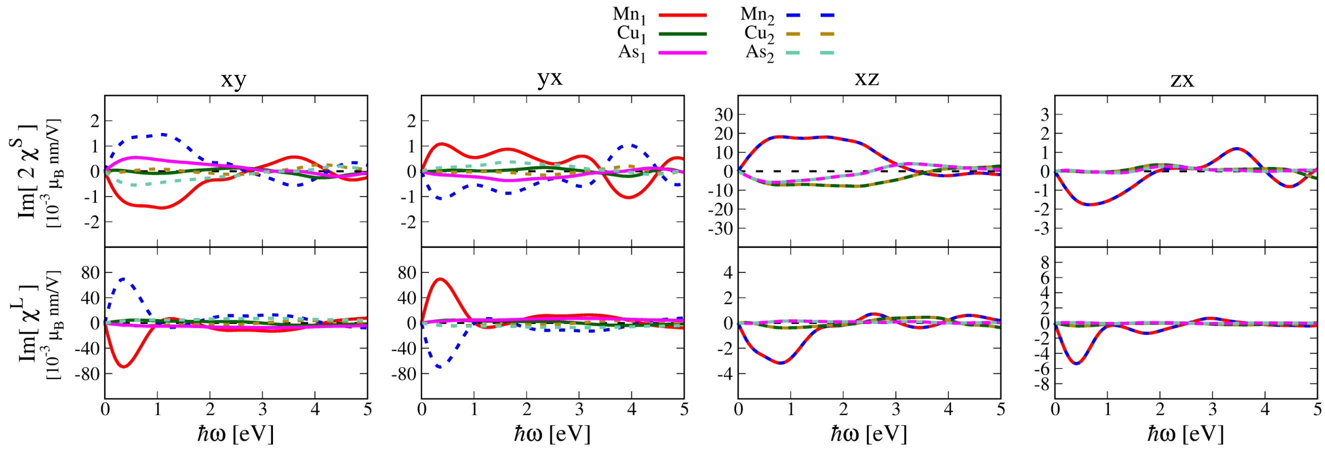

Supplementary Figure 2: Rashba-Edelstein tensors for antiferromagnetic CuMnAs with moments along the a-axis. Sown are the calculated imaginary parts of the nonzero elements of the spin and orbital Rashba-Edelstein effect tensors, $2\chi^S_{ij}(\omega)$ and $\chi^L_{ij}(\omega)$, i, j = x, y or z.

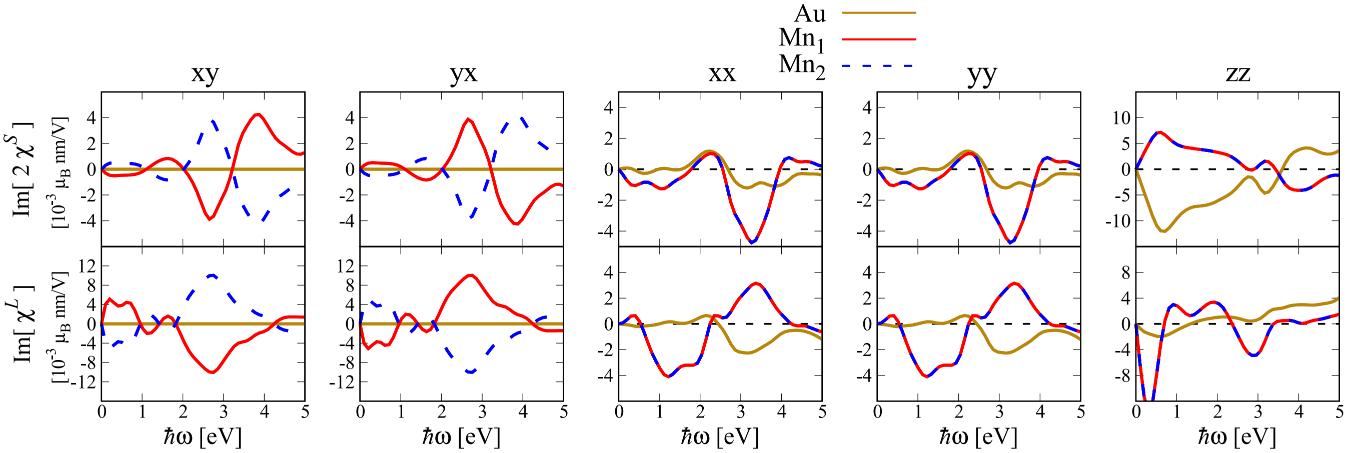

Supplementary Figure 3: Rashba-Edelstein tensors for antiferromagnetic Mn_2Au with moments along the c-axis. Sown are the calculated imaginary parts of the nonzero elements of the spin and orbital Rashba-Edelstein effect tensors, $2\chi^S_{ij}(\omega)$ and $\chi^L_{ij}(\omega)$, i, j = x, y or z.

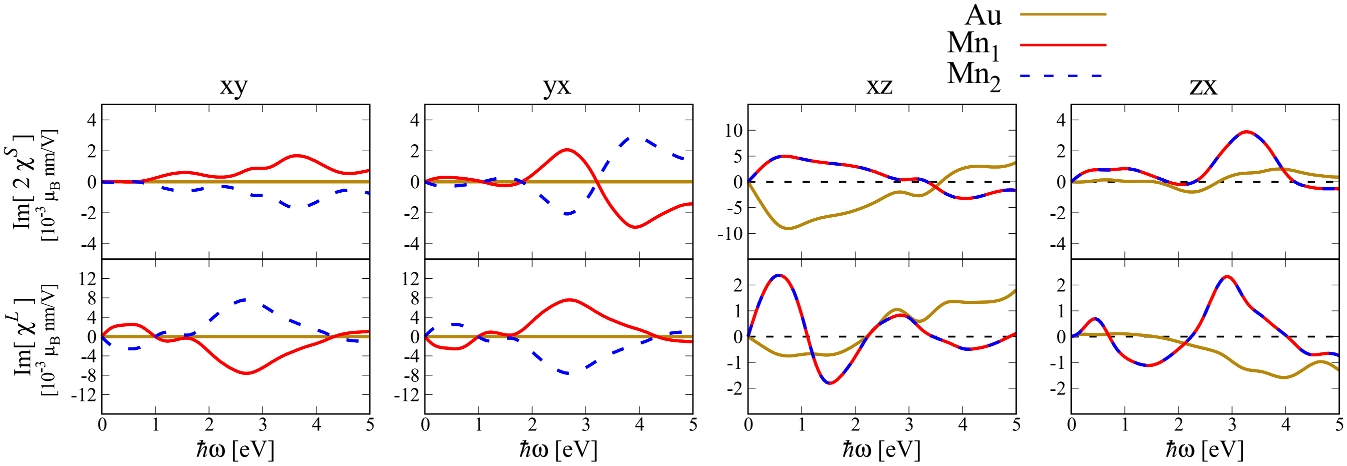

Supplementary Figure 4: Rashba-Edelstein tensors for antiferromagnetic Mn_2Au with moments along the a-axis. Sown are the calculated imaginary parts of the nonzero elements of the spin and orbital Rashba-Edelstein effect tensors, $2\chi^S_{ij}(\omega)$ and $\chi^L_{ij}(\omega)$, i, j = x, y or z.

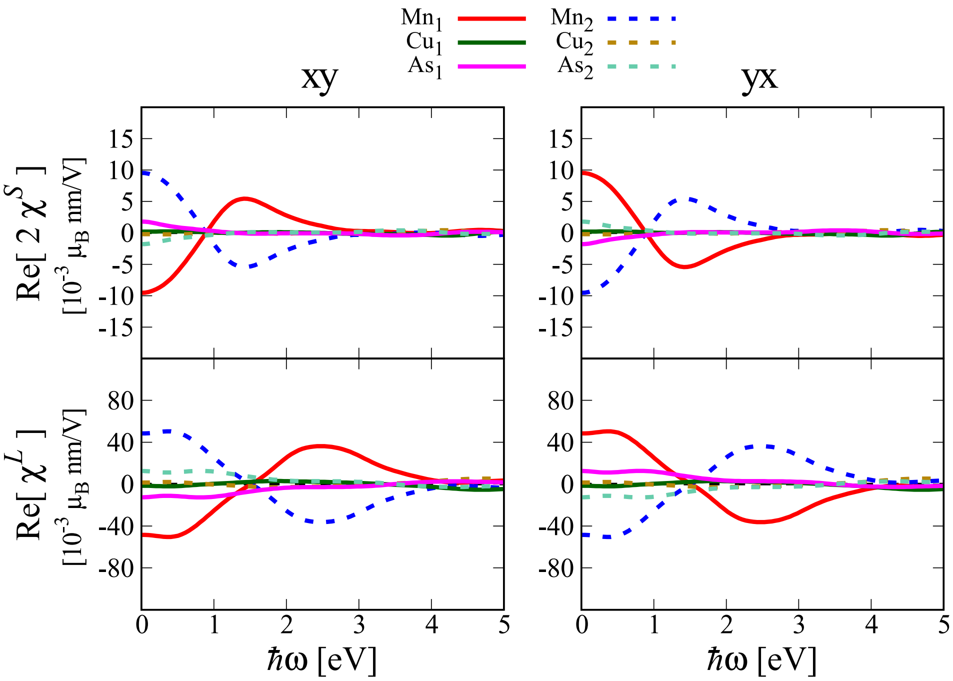

Supplementary Figure 5: Rashba-Edelstein effect of nonmagnetic tetragonal CuMnAs. Shown are the calculated real parts of the nonzero elements of the spin and orbital Rashba-Edelstein effect tensors, $2\chi^S_{ij}(\omega)$ and $\chi^L_{ij}(\omega)$.

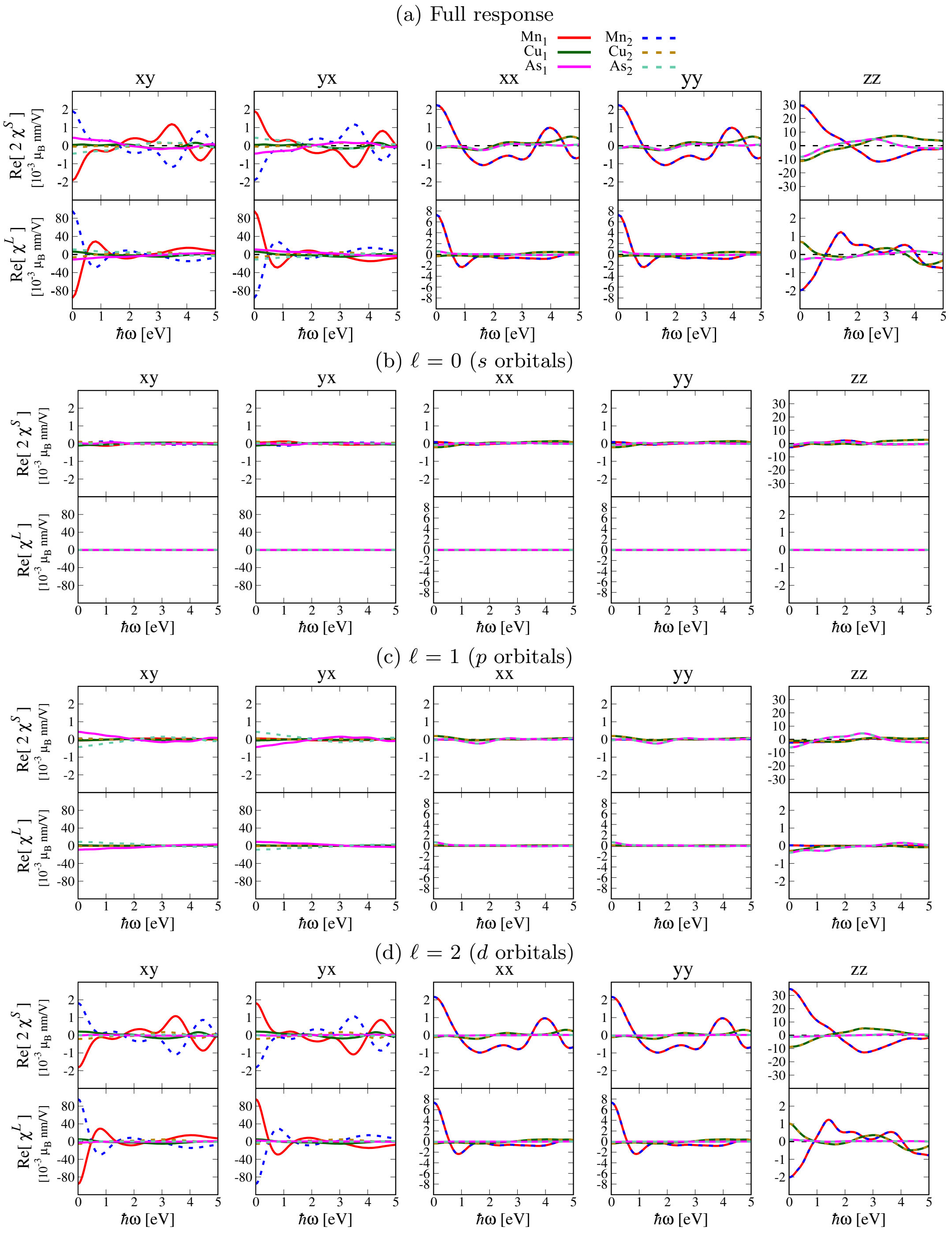

Supplementary Figure 6: Orbital-projected spin and orbital Rashba-Edelstein effect of CuMnAs. The panels correspond, respectively, to (a) the full response, (b) the response of the s orbitals, (c) response of the p orbitals, and (d) response of the d orbitals. The Rashba-Edelstein susceptibility tensors for the Mn atoms, which give the largest response, arise mainly from the d electrons. The results shown are calculated for antiferromagnetic CuMnAs with magnetic moments along the c-axis.

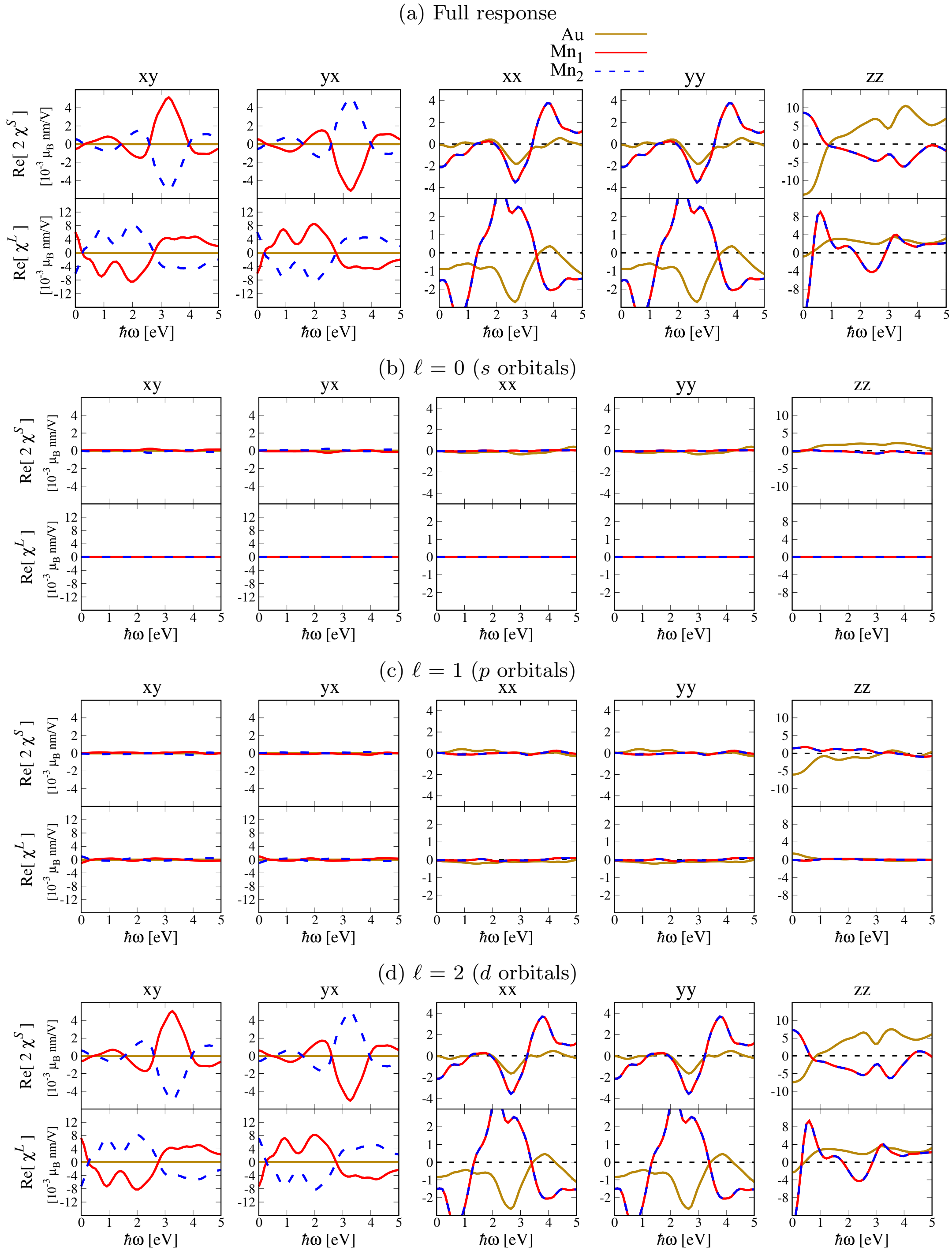

Supplementary Figure 7: Orbital-projected spin and orbital Rashba-Edelstein effect of Mn_2Au. The panels correspond, respectively, to (a) the full response, (b) the response of the s orbitals, (c) response of the p orbitals, and (d) response of the d orbitals. The Rashba-Edelstein susceptibility tensors for the Mn atoms, which give the largest response, arise mainly from the d electrons. The results shown are calculated for antiferromagnetic Mn_2Au with magnetic moments along the c-axis.

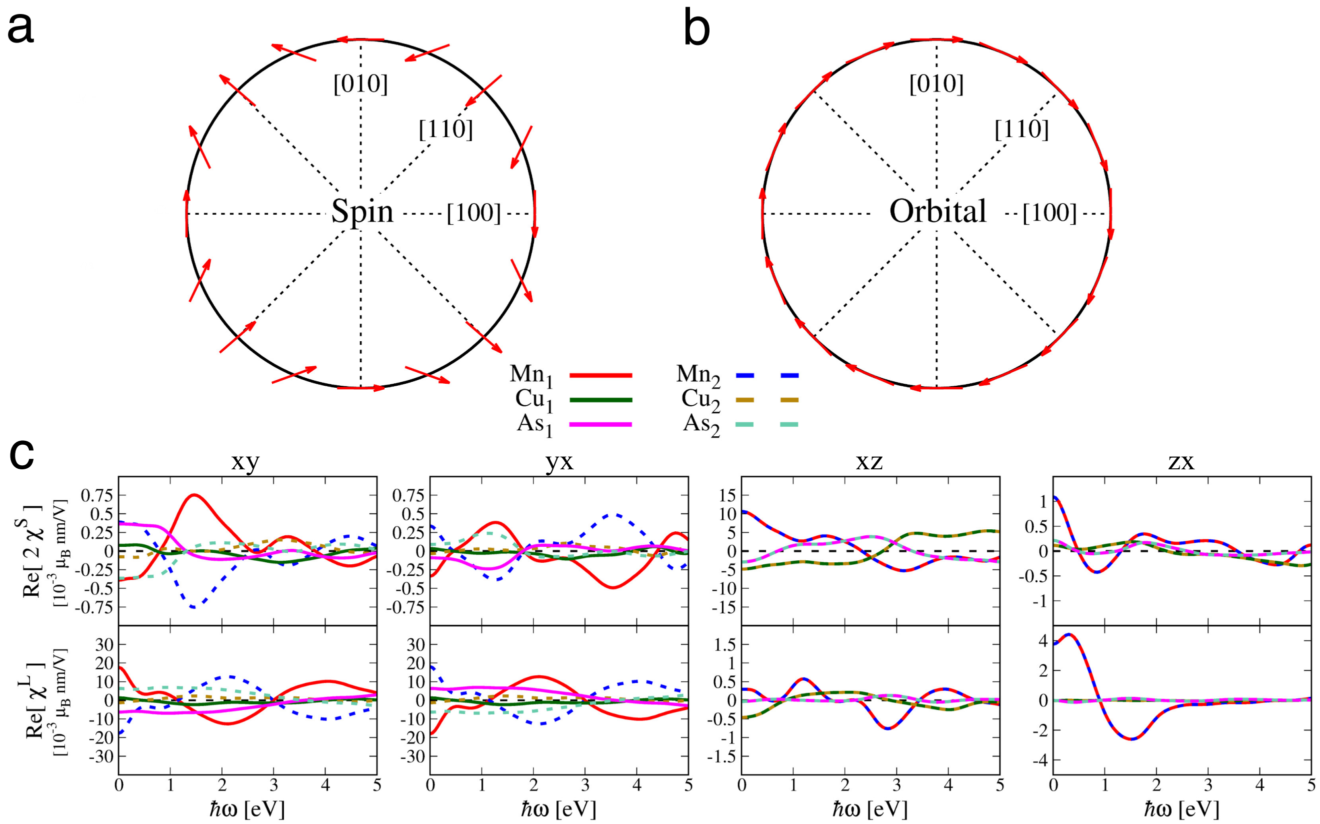

Supplementary Figure 8: Influence of a chemical potential shift on the Rashba-Edelstein susceptibility tensors. Shown are results calculated for antiferromagnetic CuMnAs with moments along the a-axis and a shift of the chemical potential by +0.6 eV. Panels (a) and (b) show the texture of the induced spin and orbital magnetization, respectively, as function of the in-plane static electric field direction for Mn_1. Panel (c) shows the calculated real parts of the nonzero elements of the spin and orbital Rashba-Edelstein tensors, $2\chi^S_{ij}(\omega)$ and $\chi^L_{ij}(\omega)$.

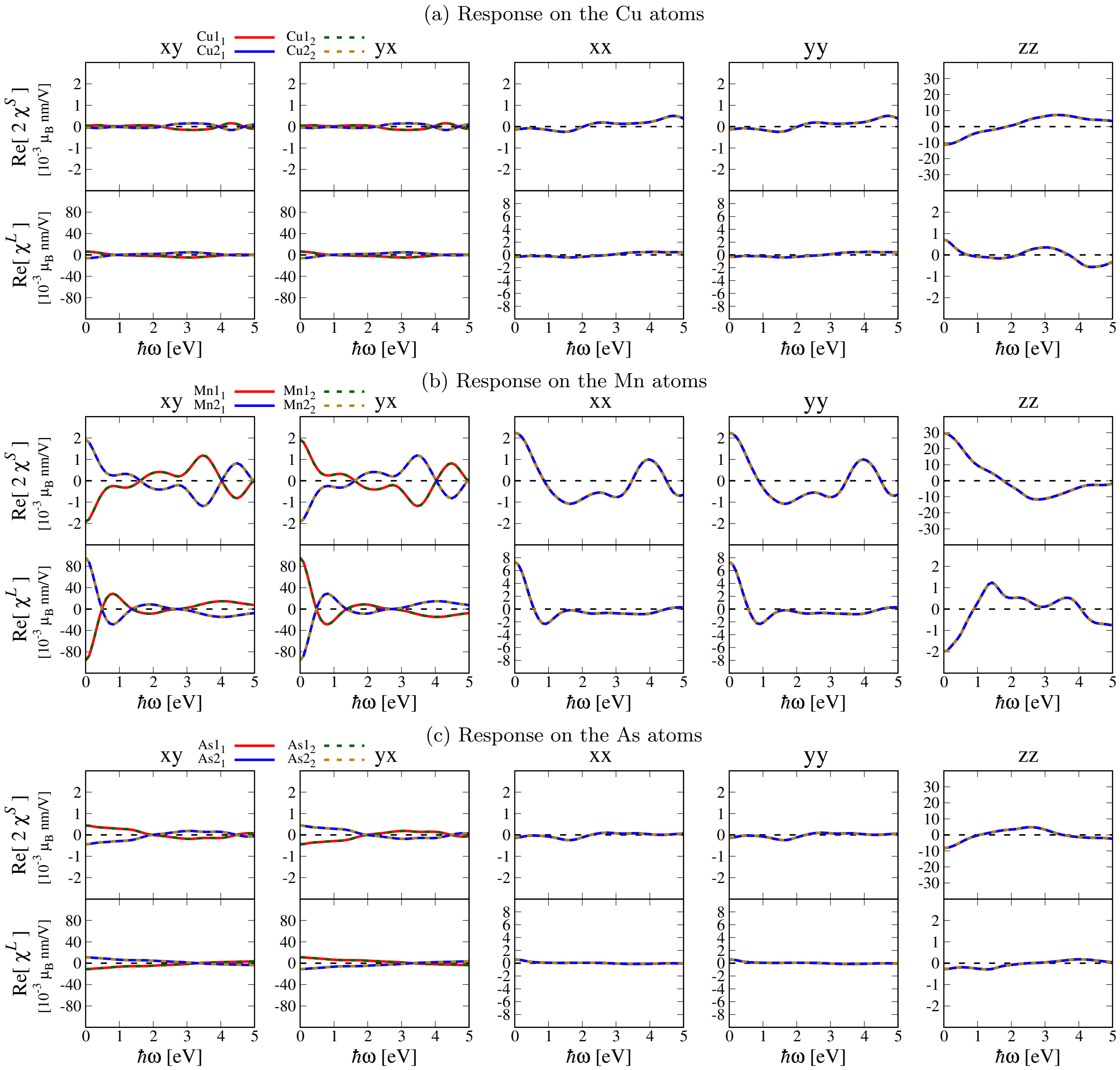

Supplementary Figure 9: Influence of unit-cell doubling on the computed Rashba-Edelstein tensors. Shown are the real parts of the spin and orbital Rashba-Edelstein tensors, $2\chi^S_{ij}(\omega)$ and $\chi^L_{ij}(\omega)$, for antiferromagnetic CuMnAs with moments along the c-axis. The computed atom-projected nonzero tensor elements are given for the different constituting atoms. (a) Response on the Cu atoms, (b) response on the Mn atoms, and (c) response on the As atoms. The atoms in the single unit cell are denoted by the subscript 1 and those in the double unit cell by subscript 2. The calculations evidence the full numerical convergence of the tensor elements with respect to the unit-cell size.
